# Effects of Strengthening‐Based Home Exercise Program on Pain and Function in Knee Osteoarthritis: Phase I Results of a Two‐Phase Randomized Controlled Trial

**DOI:** 10.1002/hsr2.72705

**Published:** 2026-06-28

**Authors:** Kazi Md Azman Hossain, Jannatul Ferdous Rikti, Md. Feroz Kabir, Sharmila Jahan, Ehsanur Rahman, K. M. Amran Hossain, Md. Kabir Hossain, Abid Hasan Khan, Suraiya Yesmin Sharna, Md. Zahid Hossain

**Affiliations:** ^1^ Department of Physiotherapy and Rehabilitation Jashore University of Science and Technology (JUST) Jashore Bangladesh; ^2^ Department of Physiotherapy BRB Hospitals Limited Dhaka Bangladesh

**Keywords:** function, home exercise, knee osteoarthritis, pain, strengthening exercise

## Abstract

**Background and Aims:**

Knee osteoarthritis (KOA) is a major cause of pain, disability, and reduced quality of life worldwide. Exercise therapy is widely recommended as a first‐line non‐pharmacological treatment; however, evidence on a strengthening‐based home exercise program (SHEP) remains limited. This study aimed to investigate the effects of 8 weeks SHEP intervention on pain and function in KOA.

**Methods:**

This randomized controlled trial involved 247 KOA participants aged 45–70, who were randomly assigned to the experimental group receiving SHEP with lifestyle advice, or the control group receiving pain medication with lifestyle advice for 8 weeks. Primary outcomes included pain intensity measured using the Numeric Pain Rating Scale (NPRS), pressure pain threshold (PPT) assessed with an algometer, and functional ability measured using the Western Ontario and McMaster Universities Osteoarthritis Index (WOMAC). Secondary outcomes included muscle strength, range of motion (ROM), Six‐Minute Walk Test (6MWT), and quality‐of‐life measures. Outcomes were assessed at baseline and after 8 weeks.

**Results:**

After 8 weeks, the experimental group demonstrated significantly greater improvements than the control group (*p* < 0.001). Pain intensity was reduced (mean difference [MD] = –1.95; 95% CI: –2.28 to –1.62), PPT increased (MD = 0.46; 95% CI: 0.32–0.60), and WOMAC total score improved (MD = –12.62; 95% CI: –14.37 to –10.86). Significant between‐group improvements were also observed in muscle strength, ROM, 6MWT, and quality‐of‐life scores (*p* < 0.001). No serious adverse events were reported throughout the trial.

**Conclusion:**

Eight weeks SHEP intervention was associated with significant improvements in pain, physical function, and quality of life in individuals with KOA. This intervention appeared to be feasible and was generally well tolerated by participants, supporting its potential as a sustainable rehabilitation strategy, particularly in resource‐limited healthcare settings.

**Trial Registration:** ID: CTRI/2025/03/081575.

## Summary

- SHEP intervention significantly improves pain, physical function, and quality of life in KOA.
- Compared with pain medication along with lifestyle advice, SHEP intervention shows superior and clinically meaningful effects.
- Feasible and low‐cost, SHEP intervention may provide an effective rehabilitation strategy in resource‐limited settings.

**Abbreviations**
6MWT: Six‐minute walk test
BMI: body mass index
CCI: Charlson Comorbidity Index
CEP: Center‐based exercise program
CG: Control group
CONSORT: Consolidated Standards of Reporting Trials
EG: Experimental group
ICC: Intraclass correlation coefficient
JUST: Jashore University of Science and Technology
KOA: Knee osteoarthritis
MCID: Minimal clinically important difference
MD: Mean difference
NPRS: Numeric pain rating scale
NSAIDs: Non‐steroidal anti‐inflammatory drugs
OARSI: Osteoarthritis Research Society International
PPT: Pressure pain threshold
RCT: Randomized controlled trial
ROM: Range of motion
SHEP: Strengthening‐based home exercise program
SPSS: Statistical Package for the Social Sciences
WOMAC: Western Ontario and McMaster Universities Osteoarthritis Index

## 1 Introduction

Osteoarthritis (OA) is a leading cause of disability worldwide, affecting an estimated 595 million individuals in 2020, representing 7.6% of the global population and marking a 132.2% increase since 1990 [1]. Knee osteoarthritis (KOA) is the most prevalent form, primarily affecting adults aged 65 and older. Radiographic evidence suggests that approximately 30% of adults aged 45 and older have KOA, while around 50% report knee pain [2, 3]. In Bangladesh, the prevalence of KOA is 7.3%, with a higher incidence among women (4.8%) compared to men (2.8%) [4, 5]. Risk factors for KOA include abnormal joint morphology, dysplasia, malalignment, muscle weakness, joint injuries, and labral tears, along with age, gender, weight, genetics, ethnicity, and diet [6, 7]. KOA commonly leads to structural and functional deterioration of articular and non‐articular tissues, manifesting as pain, restricted joint mobility, altered gait, impaired balance and proprioception, muscle weakness, crepitus, episodic effusion, and reduced overall quality of life. These limitations may reduce physical activity, contributing to depression, fatigue, disability, decreased productivity, and increased healthcare costs [1, 6, 7, 8]. Given the significant personal and societal burden of KOA, interventions that prevent symptom progression and restore function are critical.

Non‐pharmacological interventions, particularly exercise, are strongly recommended for KOA management by international guidelines, including the Osteoarthritis Research Society International (OARSI) [9]. Exercise programs may be delivered through a center‐based exercise program (CEP) or a home‐based exercise program (HEP), both of which have demonstrated efficacy in improving pain, physical function, and quality of life compared with no intervention or standard care [3]. HEP offers a convenient, flexible, and low‐cost alternative to CEP, especially during situations that limit healthcare access, such as the COVID‐19 pandemic, or for patients facing transportation or financial barriers. Previous HEP‐based studies incorporated strengthening, stretching, aerobic, balance, and proprioceptive exercises to improve pain control, functional ability, and quality of life in KOA [10]. However, existing studies show considerable variation in exercise type, intensity, frequency, and progression, as well as small sample sizes, low‐quality evidence, high risk of bias, non‐standard diagnostic criteria, and related factors that limit their generalizability across diverse populations [3, 10, 11]. Multiple international clinical practice guidelines recommend strengthening exercises for managing KOA. But there is a lack of a well‐structured SHEP intervention, even though strengthening exercises are considered the most important type for KOA. These exercises can increase muscle strength around the knee joint, improve joint stability, and reduce pain from mechanical stress in KOA [12]. However, the limited number of high‐quality studies emphasizes the need for carefully designed, culturally relevant, and well‐structured SHEP intervention.

Additionally, previous studies suggest that the benefits of exercise may diminish over time, underscoring the need for interventions that patients can safely continue independently after program completion [11]. Furthermore, despite the potential benefits of HEP in terms of accessibility and cost‐effectiveness, evidence regarding their feasibility, adherence, and impact on multiple outcomes in KOA within the South Asian context remains limited [3, 13]. However, to our knowledge, no studies have directly compared evidence‐based SHEP intervention with medication‐based care. Addressing these gaps is essential for informing clinical practice and developing sustainable, community‐based rehabilitation strategies that reduce the burden of KOA while empowering patients to manage their condition effectively.

Considering the limited availability of high‐quality, conclusive evidence supporting the effectiveness of SHEP intervention and the uncertainty regarding the most effective HEP type for KOA, we designed a two‐phase randomized controlled trial (RCT). This first‐phase trial evaluates the effects of SHEP intervention using a rigorous methodological design. Therefore, the primary objective of this RCT was to investigate the effects of 8 weeks SHEP intervention on pain and function in participants with KOA. The secondary objectives were to (1) assess the baseline compatibility between groups; (2) find out the contributing sociodemographic characteristics of KOA; (3) investigate the effectiveness of SHEP intervention versus pain medication on pain, physical function, muscle strength, range of motion, walking ability, and quality of life in KOA. This first phase was based on a two‐tailed hypothesis. It was assumed that SHEP intervention or pain medication could lead to superior improvements in outcome measurements for KOA. As the study population was drawn from the community, most participants had a low level of education, raising concerns about the proper execution of exercises without supervision. Although strict methodological rigor was maintained to achieve optimal outcomes, the possibility of varied results persisted, contributing to uncertainty and thus supporting the use of a two‐tailed hypothesis.

## 2 Methods

### 2.1 Study Overview

**Figure 1:**
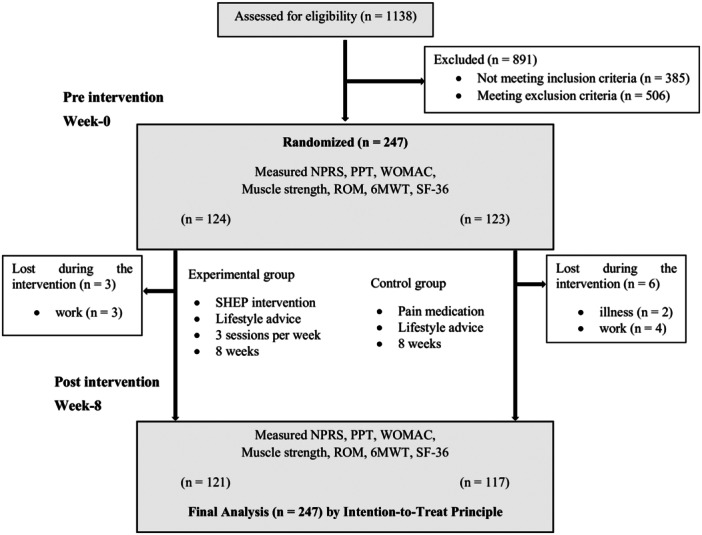
Study flow diagram. 6MWT., 6‐Minute Walk Test; NPRS, Numeric Pain Rating Scale; PPT, Pressure Pain Threshold; ROM, Range of Motion; SF‐36, Short Form Survey‐36; SHEP, Strengthening‐based Home Exercise Program; WOMAC, Western Ontario and McMaster Universities Osteoarthritis Index.

This assessor‐blinded, parallel‐group RCT was conducted in Bangladesh from April to December 2025 and included 247 participants with KOA. Participant eligibility screening and recruitment were conducted using online and offline advertisements to reach the target population. Eligible participants were consecutively enrolled, and baseline assessments were completed immediately upon enrollment. Participants were then allocated in a 1:1 ratio, according to a pre‐specified randomization sequence, to either the experimental group (EG), receiving SHEP intervention along with lifestyle advice, or the control group (CG), receiving pain medication along with lifestyle advice. Both groups underwent 8 weeks intervention, with post‐intervention assessments conducted sequentially as participants completed the program. This approach ensured efficient participant flow, standardized intervention delivery, and high‐quality data collection within the planned study period. All resources and logistics support are provided by the Department of Physiotherapy and Rehabilitation at Jashore University of Science and Technology (JUST), Jashore, Bangladesh. The study was ethically approved, prospectively registered with the WHO Clinical Trial Registry, and the protocol was published [13]. Reporting adhered to the Consolidated Standards of Reporting Trials (CONSORT) guidelines (Figure 1).

### 2.2 Participants

A total of 11 health awareness programs and discussion sessions were arranged to get the required participants through offline and online advertisements. Participants were recruited in accordance with the study's eligibility criteria. Participants were included if they were: (1) aged 45–70 years for both genders; (2) diagnosed with KOA for a minimum of 6 months according to American College of Rheumatology guidelines [14]; (3) capable of mobility without assistive devices and knee flexion > 90° [15]; (4) radiographs showing Kellgren and Lawrence Grade II or III KOA [16]; (5) having access to devices for viewing video tutorials. Participants were excluded if they: (1) had undergone knee surgery in the prior 12 months [15]; (2) presented with lumbar radiculopathy, vascular claudication, severe anterior knee pain due to patella‐femoral syndrome or chondromalacia, or any additional neurological, muscular, or joint conditions that could hinder lower limb function [15, 17, 18]; (3) visible malformation of the knee, characterized as either varus or valgus [18]; (4) received hyaluronic acid or corticosteroid injections within 3 months prior to study enrollment [15]; (5) current pregnancy; (6) BMI above 30 to minimize confounding from obesity‐related biomechanical loading, metabolic inflammation, and functional heterogeneity that may independently affect treatment response and exercise tolerance in KOA [19].

### 2.3 Intervention

Two groups were formed from eligible participants. The experimental group completed 8 weeks SHEP intervention along with lifestyle advice, guided by four experienced musculoskeletal physiotherapists (> 7 years' experience). Each participant was provided with three resistance TheraBands (yellow, red, green), a printed “Health Knowledge and Home Exercise Guide,” and a Bengali‐language instructional video. The program consisted of 3 weekly sessions, each beginning with a 5‐min warm‐up (light walk and march) followed by joint mobility exercises. Strengthening exercises included TheraBand‐based knee flexion–extension, terminal knee extension, leg press, calf raises, and mini‐squats, performed with progression in resistance and dosage: Stage 1 (sessions 1–8)—3 sets of 10 repetitions with the yellow band; Stage 2 (sessions 9–16)—3 sets of 15 repetitions with the red band; and Stage 3 (sessions 17–24)—3 sets of 20 repetitions with the green band. Each set was separated by 15 s of rest, and different exercise modes by 1 min of rest. Sessions concluded with a 5‐min cool‐down of static stretching (hip flexor, outer hip, quadriceps, hamstrings; 3 repetitions, 15‐second holds). The detailed exercise protocol is also provided in Supporting Information S1 [20, 21].

Participants in the control group received pain medication—non‐steroidal anti‐inflammatory drugs (NSAIDs) for symptomatic management, specifically ibuprofen 400 mg three times daily or naproxen 250 mg twice daily, prescribed according to symptom severity. In addition, participants were provided with a printed “Health Knowledge and Advice Guide” in Bengali that included general healthy‐lifestyle recommendations, such as daily walking, knee‐joint protection strategies, and weight management. No additional co‐interventions, such as physiotherapy sessions or other analgesics, were initiated during the trial, minimizing potential confounding effects. Both groups received twice‐weekly telephone calls and follow‐up messages for motivation and compliance. Adherence to the progression was monitored through participants' logs and researcher checklists that recorded session frequency, duration, and approach [13].

### 2.4 Intervention Fidelity and Adherence Monitoring

Intervention fidelity was ensured through standardized delivery across four experienced musculoskeletal physiotherapists, who completed a structured orientation covering exercise prescription, progression, safety monitoring, and communication protocols. Consistency was further supported using a standardized exercise manual, identical Bengali‐language instructional videos, and uniform TheraBand resistance levels. For participants in the experimental group, the first session of each week was directly supervised by trained personnel through home visits. During these visits, exercise performance was monitored, and participants received direct guidance to ensure proper execution and minimize exercise‐related errors. Additionally, adherence to the SHEP intervention was tracked via participants' completed logs and reinforced through twice‐weekly telephone calls and follow‐up messages. Control group participants' medication use and lifestyle were also monitored through self‐report logs and weekly follow‐up calls. Among the 247 enrolled participants, 238 completed the trial (attrition: *n *= 9). Overall adherence among completers was high. In the experimental group (*n *= 121), mean adherence to the prescribed 24‐session exercise program was 98% (percentage of prescribed exercise sessions completed). In the control group (*n *= 117), adherence to prescribed medications and lifestyle advice was estimated at 93.5%, based on self‐reported compliance and monitoring records. (Figure 1). This combination of direct and indirect monitoring ensured proper implementation of the study intervention and minimized errors in exercise performance.

### 2.5 Outcome Measurements

Outcome measurements included primary and secondary outcome measures including sociodemographic factors such as age, gender, BMI (body mass index), KOA severity by radiographic grading, duration of symptoms, previous fall episodes, presence of comorbidities (e.g., hypertension and diabetes mellitus) as measured by the Charlson Comorbidity Index (CCI), and physical activity or work status were included as generalized determinants, all measured at baseline. The primary and secondary outcomes were measured at baseline and at post‐intervention (8 weeks) by trained, blinded assessors.

#### 2.5.1 Primary Outcomes

Primary outcomes included pain intensity, pressure pain threshold (PPT), and physical function. Pain intensity was assessed using the Numerical Pain Rating Scale (NPRS; 0 = no pain, 10 = worst pain), which has previously demonstrated high reliability (ICC = 0.95) [22]. PPT was measured with a hand‐held pressure algometer (FPK 20, Wagner Instruments, USA) at the most tender knee site, recorded three times at 30‐second intervals, and averaged. It's a reliable method for measuring pain sensitivity (ICC = 0.91) [23]. Physical function was evaluated using the Western Ontario and McMaster University Osteoarthritis Index (WOMAC), comprising 24 items across pain, stiffness, and function subscales (ICC = 0.86, 0.68, and 0.89, respectively) [24].

#### 2.5.2 Secondary Outcomes

Secondary outcomes included muscle strength, range of motion (ROM), walking ability, and quality of life. Muscle strength was assessed with a modified sphygmomanometer for knee flexor and extensor muscles (0–300 mmHg). It provides a valid, safe, and low‐cost approach for assessing muscle strength, with excellent reliability (ICC = 0.80–0.99) [25, 26]. ROM was measured using a long‐arm universal goniometer for knee flexion–extension (ICC = 0.99) [27]. Walking ability was evaluated by the 6‐min Walk Test (6MWT) in a 24‐meter corridor (ICC = 0.86–0.96) [28]. Quality of life was measured using the 36‐item Short Form Survey (SF‐36) across eight domains; the mean score across these domains was reported, with higher scores indicating better health status (ICC = 0.90) [29].

### 2.6 Sample Size

The sample size was determined using G*Power version 3.1.9.7 (University of Kiel, Kiel, Germany), based on the primary outcome, physical function. An effect size (ES) of 0.41 was assumed, derived from previously reported improvements in function among individuals with KOA [30]. The calculation used a two‐tailed significance level of *α* = 0.05 and a statistical power of 80%, yielding a required sample of 190 participants. To maintain adequate power despite potential attrition, a conservative 30% dropout rate was assumed, resulting in a recruitment target of 247 participants. This higher‐than‐typical rate posed challenges for monitoring SHEP intervention adherence and safeguarded the internal validity of Phase I. After enrolling 247 eligible participants who provided informed consent, recruitment was discontinued.

### 2.7 Randomization and Blinding

After obtaining written informed consent, eligible participants were enrolled consecutively and randomly assigned in a 1:1 ratio to either the experimental or control group using a computer‐generated randomization sequence created with the Microsoft Excel RAND() function. The sequence was generated by two independent research assistants who were not involved in participant recruitment, baseline assessment, intervention delivery, or outcome evaluation, thereby minimizing the risk of selection bias. Prior to allocation, the randomization list was stratified by age and gender to ensure balance across these key prognostic variables; no blocking or fixed block sizes were applied, and simple stratified randomization was used. Allocation concealment was ensured using sequentially numbered, opaque, sealed envelopes (SNOSE) prepared and verified by an independent coordinator not involved in participant handling; envelopes were identical, tamper‐proof, and opened sequentially only after completion of all baseline assessments. To enhance transparency and reproducibility, all steps of sequence generation, stratification, and envelope preparation were fully documented, independently cross‐checked, and securely archived. Participants were aware of their assigned intervention due to the nature of the programs; however, study hypotheses and expected group superiority were not disclosed to reduce performance bias. Treating health professionals and two research assistants were not blinded, whereas all outcome assessments were conducted by trained, blinded assessors following standardized procedures to maintain blinding. Data analysis was performed by a blinded statistician, and independent trial monitoring staff ensured adherence to randomization, allocation concealment, and blinding procedures throughout the study.

### 2.8 Statistical Analysis

Data were analyzed using SPSS version 26.0 (IBM, Armonk, NY, USA). Continuous variables were presented as mean ± standard deviation (SD), and categorical variables as frequency and percentages. Normality of variables was assessed using the Shapiro–Wilk test. Between‐group differences at baseline and post‐intervention were analyzed using an independent *t*‐test, with results reported as mean ± SD, mean differences (MD) with 95% confidence intervals (CI), *t*‐values, and *p* values. Cohen's *d* was reported to estimate between‐group effect size. Repeated‐measures ANOVA was used to assess within‐group changes over time, between‐group differences, and time‐by‐group interactions, with results reported as *F* value, *p* value, and partial eta squared (*ηp* ^2^) as a measure of effect size. Where significant interaction effects were observed, post hoc pairwise comparisons of estimated marginal means were conducted with Bonferroni adjustment to control for multiple testing across outcome measures. Bonferroni adjustment was applied only to post hoc pairwise comparisons within individual outcomes. No correction was applied across multiple outcome measures; therefore, the potential for inflated family‐wise Type I error should be considered when interpreting the results. As the study included only two repeated measurements (baseline and Week 8), the assumption of sphericity was inherently satisfied; therefore, Mauchly's test and related corrections were not required. Analyses followed the intention‐to‐treat principle, including all 247 randomized participants. Missing data were handled using multiple imputation under a missing‐at‐random assumption. Fully conditional specification (FCS) was used to generate 20 imputed data sets, incorporating baseline demographic variables, group allocation, and all repeated outcome measures in the imputation model. Pooled estimates were derived according to Rubin's rules. Statistical significance was set at *p* < 0.05, *p* < 0.01, and *p* < 0.001 [31].

### 2.9 Participants and Public Involvement

Participants or the public were not involved in the design, conduct, or reporting of the trial.

### 2.10 Ethical Considerations

This study was approved by the Institutional Review Board of the Department of Physiotherapy and Rehabilitation, Jashore University of Science and Technology, on January 07, 2025 (IRB ID: PTR‐JUST/IRB/2025/02/192404). The study was also prospectively registered with the Clinical Trial Registry of India on March 03, 2025 (ID: CTRI/2025/03/081575). Written informed consent was obtained from all participants prior to enrollment. All procedures and interventions were conducted in accordance with the principles outlined in the Declaration of Helsinki (2020).

## 3 Results

### 3.1 Participant Flow and Baseline Characteristics

**Figure 2:**
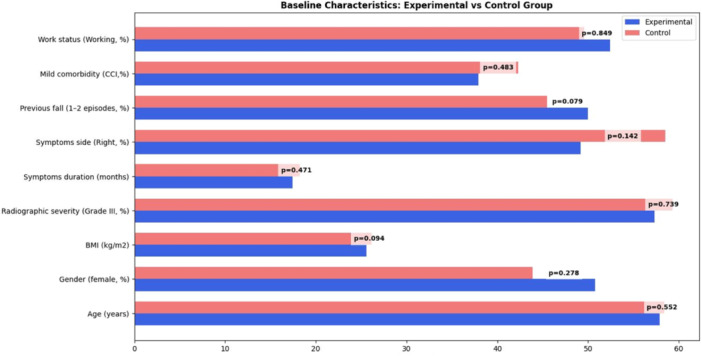
Baseline characteristics: experimental vs. control group.

A total of 247 participants were enrolled in this study; however, 9 did not complete the post‐intervention evaluation, including 3 from the experimental group and 6 from the control group, due to their work and other illnesses (Figure 1). The experimental and control groups were well balanced at baseline across all demographic, clinical, and functional characteristics, supporting the internal validity of subsequent comparisons (Table 1). The mean age was almost similar between groups (57.90 ± 6.91 years in the experimental group vs. 58.42 ± 6.81 years in the control group), with a comparable gender distribution (50.8% females in the experimental group vs. 43.9% in the control group). Body mass index was also comparable (25.58 ± 2.61 kg/m^2^ vs. 26.14 ± 2.65 kg/m^2^). Regarding disease‐specific characteristics, the majority of participants had moderate radiographic severity (Grade III: 57.3% vs. 59.3%), with comparable mean symptom duration (17.43 ± 8.85 months vs. 18.26 ± 9.29 months) and a slightly higher proportion of right‐side involvement in the control group (58.5% vs. 49.2%). Fall history, comorbidity burden, and work status were similarly distributed, with most participants reporting 1–2 recent falls, mild comorbidities, and active employment. These distributions demonstrate that the randomization process produced comparable groups, ensuring that observed post‐intervention differences are attributable to the interventions rather than baseline imbalances. Figure 2 highlights the visual aspect of participants' baseline characteristics comparison between groups.

**Table 1:** Participants' baseline characteristics.

Characteristics	EG (*n* = 124)	CG (*n* = 123)	Total (*n* = 247)
Age (years), mean (SD)	57.90 (6.91)	58.42 (6.81)	58.16 (6.85)
Gender, *n* female (%)	63 (50.8)	54 (43.9)	117 (47.4)
Body mass index (kg/m^2^), mean (SD)	25.58 (2.61)	26.14 (2.65)	25.86 (2.64)
Radiographic severity (grade), *n* (%)			
Grade II	53 (42.7)	50 (40.7)	103 (41.7)
Grade III	71 (57.3)	73 (59.3)	144 (58.3)
Symptoms duration (months), mean (SD)	17.43 (8.85)	18.26 (9.29)	17.84 (9.06)
Symptoms side, *n* right (%)	61 (49.2)	72 (58.5)	133 (53.8)
Previous fall episode, *n* (%)			
No fall (0)	35 (28.2)	47 (38.2)	82 (33.2)
Fall history (1–2)	62 (50.0)	56 (45.5)	118 (47.8)
Fall history (3–4)	23 (18.5)	18 (14.6)	41 (16.6)
Fall history (≥ 5)	4 (3.2)	2 (1.6)	6 (2.4)
Comorbidity (based on CCI), *n* (%)			
No comorbidity	52 (41.9)	45 (36.6)	97 (39.3)
Mild comorbidity	47 (37.9)	52 (42.3)	99 (40.1)
Moderate comorbidity	21 (16.9)	22 (17.9)	43 (17.4)
Severe comorbidity	4 (3.2)	4 (3.3)	8 (3.2)
Work status, *n* (%)			
Working	65 (52.4)	61 (49.6)	126 (51.0)
Not working	39 (31.5)	45 (36.6)	84 (34.0)
Retired	20 (16.1)	17 (13.8)	37 (15.0)
Unknown	0	0	0

Abbreviations: CG, control group; EG, experimental group.

### 3.2 Changes in Outcome Measures Over Time

**Figure 3:**
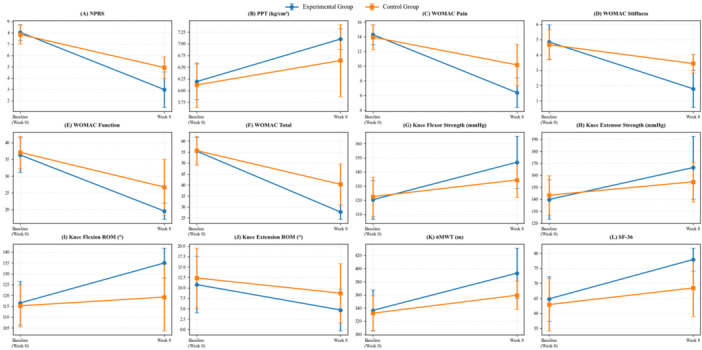
Changes in outcome measurements. *Note:* Mean ± SD values for primary and secondary outcomes at baseline and Week 8 in the experimental and control groups. Error bars indicate standard deviations.

Based on Tables 2 and 3, repeated‐measures ANOVA revealed highly significant time effects across all primary and secondary outcomes (all *p* < 0.001), with large effect sizes (*ηp* ^2^ = 0.362–0.883), indicating substantial improvements over the 8‐week intervention period in both groups. Significant group effects were also observed for all outcomes (*p* < 0.05), with effect sizes ranging from small to moderate (*ηp* ^2^ = 0.019–0.306), demonstrating overall superiority of the experimental group. More importantly, significant group × time interaction effects were identified for every outcome (all *p* < 0.001), with interaction effect sizes ranging from moderate to large (*ηp* ^2^ = 0.054–0.350), confirming that the magnitude of improvement over time was consistently greater in the experimental group than in the control group. No statistically significant between‐group differences were found at baseline for any outcome, as evidenced by confidence intervals crossing zero and non‐significant *p* values, indicating comparable groups prior to intervention. At post‐intervention, however, the experimental group demonstrated significantly greater improvements across all outcomes, supported by 95% confidence intervals that excluded the null value and favored the experimental intervention. Figure 3 highlights the visual comparison of outcome measures between groups.

**Table 2:** Changes in primary outcome measures.

Variables	Group	Baseline week–0	Post‐intervention week–8	Cohen's *d*	Repeated measures ANOVA		
					*F*	*F*	*F*
					*p*	*p*	*p*
					*ηp* ^2^ (time)	*ηp* ^2^ (group)	*ηp* ^2^ (interaction)
NPRS	EG	8.05 (0.69)	2.98 (1.58)	2.79	*F* = 1851.854 *p* < 0.001 *ηp* ^2^ = 0.883	*F* = 78.571 *p* < 0.001 *ηp* ^2^ = 0.243	*F* = 131.968 *p* < 0.001 *ηp* ^2^ = 0.350
	CG	7.86 (0.84)	4.93 (0.95)				
	Between group MD (95% CI)	0.19 (–0.01 to 0.38)	–1.95 (–2.28 to –1.62)				
	*T*	1.909	–11.748				
	*p*	0.057	*p* < 0.001				
PPT, kg/cm^2^	EG	6.19 (0.38)	7.10 (0.22)	0.90	*F* = 274.396 *p* < 0.001 *ηp* ^2^ = 0.529	*F* = 32.211 *p* < 0.001 *ηp* ^2^ = 0.117	*F* = 19.396 *p* < 0.001 *ηp* ^2^ = 0.074
	CG	6.12 (0.48)	6.64 (0.77)				
	Between group MD (95% CI)	0.07 (–0.04 to 0.18)	0.46 (0.32 to 0.60)				
	*T*	1.276	6.360				
	*p*	0.203	*p* < 0.001				
WOMAC pain	EG	14.30 (1.35)	6.39 (2.02)	2.68	*F* = 1033.448 *p* < 0.001 *ηp* ^2^ = 0.808	*F* = 84.435 *p* < 0.001 *ηp* ^2^ = 0.256	*F* = 129.002 *p* < 0.001 *ηp* ^2^ = 0.345
	CG	13.95 (1.71)	10.17 (2.83)				
	Between group MD (95% CI)	0.35 (−0.04 to 0.73)	–3.78 (–4.40 to –3.17)				
	*T*	1.769	–12.110				
	*p*	0.078	*p* < 0.001				
WOMAC stiffness	EG	4.85 (1.14)	1.79 (1.22)	1.71	*F* = 704.309 *p* < 0.001 *ηp* ^2^ = 0.742	*F* = 52.433 *p* < 0.001 *ηp* ^2^ = 0.176	*F* = 128.346 *p* < 0.001 *ηp* ^2^ = 0.344
	CG (n = 123)	4.67 (0.99)	3.44 (0.62)				
	Between group MD (95% CI)	0.18 (–0.09 to 0.45)	–1.65 (–1.89 to –1.41)				
	*T*	1.326	–13.405				
	*p*	0.186	*p* < 0.001				
WOMAC function	EG	36.30 (5.16)	19.52 (2.41)	1.28	*F* = 739.445 *p* < 0.001 *ηp* ^2^ = 0.751	*F* = 60.550 *p* < 0.001 *ηp* ^2^ = 0.198	*F* = 41.176 *p* < 0.001 *ηp* ^2^ = 0.144
	CG	37.07 (4.83)	26.70 (8.41)				
	Between group MD (95% CI)	–0.78 (–2.03 to 0.48)	–7.18 (–8.73 to –5.64)				
	*T*	–1.218	–9.145				
	*p*	0.224	*p* < 0.001				
WOMAC total	EG	55.44 (6.23)	27.69 (3.23)	1.94	*F* = 1366.231 *p* < 0.001 *ηp* ^2^ = 0.848	*F* = 107.972 *p* < 0.001 *ηp* ^2^ = 0.306	*F* = 112.335 *p* < 0.001 *ηp* ^2^ = 0.314
	CG	55.69 (6.50)	40.31 (9.36)				
	Between group MD (95% CI)	–0.25 (–1.84 to 1.35)	−12.62 (–14.37 to –10.86)				
	*T*	–0.305	–14.189				
	*p*	0.760	*p* < 0.001				

*Note:* Independent‐*t*‐test; repeated measures ANOVA.

Values are presented as mean (SD); *p < 0.05; **p < 0.01; ***p < 0.001.

Abbreviations: CG, control group; EG, experimental group; MD, mean differences; NPRS, numeric pain rating scale; PPT, pressure pain threshold; WOMAC, Western Ontario and McMaster Universities Osteoarthritis. Index.

**Table 3:** Changes in secondary outcome measures.

Variables	Group	Baseline week–0	Post‐intervention week–8	Cohen's *d*	Repeated measures ANOVA		
					*F*	*F*	*F*
					*p*	*p*	*p*
					*ηp* ^2^ (time)	*ηp* ^2^ (group)	*ηp* ^2^ (interaction)
Knee flexor muscle strength, mmHg	EG	120.31 (13.73)	146.85 (18.38)	1.06	*F* = 317.668 *p* < 0.001 *ηp* ^2^ = 0.565	*F* = 11.246 *p* < 0.01 *ηp* ^2^ = 0.044	*F* = 47.077 *p* < 0.001 *ηp* ^2^ = 0.161
	CG	122.51 (13.91)	134.30 (12.44)				
	Between group MD (95% CI)	–2.20 (–5.66 to 1.27)	12.56 (8.62 to 16.50)				
	*T*	–1.250	6.283				
	*p*	0.213	*p* < 0.001				
Knee extensor muscle strength, mmHg	EG	139.83 (16.38)	166.43 (26.10)	0.94	*F* = 143.623 *p* < 0.001 *ηp* ^2^ = 0.370	*F* = 4.847 *p* < 0.05 *ηp* ^2^ = 0.019	*F* = 23.933 *p* < 0.001 *ηp* ^2^ = 0.089
	CG	143.39 (16.31)	154.57 (16.66)				
	Between group MD (95% CI)	–3.56 (–7.65 to 0.54)	11.87 (6.38 to 17.36)				
	*T*	–1.710	4.257				
	*p*	0.089	*p* < 0.001				
Knee flexion ROM	EG	116.53 (10.06)	134.96 (6.91)	1.49	*F* = 139.158 *p* < 0.001 *ηp* ^2^ = 0.362	*F* = 69.229 *p* < 0.001 *ηp* ^2^ = 0.220	*F* = 57.268 *p* < 0.001 *ηp* ^2^ = 0.189
	CG	115.20 (9.59)	119.23 (15.59)				
	Between group MD (95% CI)	1.33 (–1.14 to 3.79)	15.73 (12.71 to 18.75)				
	*T*	1.063	10.266				
	*p*	0.289	*p* < 0.001				
Knee extension ROM	EG	10.77 (6.78)	4.68 (5.01)	0.34	*F* = 225.229 *p* < 0.001 *ηp* ^2^ = 0.479	*F* = 13.274 *p* < 0.001 *ηp* ^2^ = 0.051	*F* = 14.000 *p* < 0.001 *ηp* ^2^ = 0.054
	CG	12.36 (7.18)	8.70 (7.10)				
	Between group MD (95% CI)	–1.59 (–3.34 to 0.16)	–4.02 (–5.56 to –2.48)				
	*T*	–1.793	–5.149				
	*p*	0.074	*p* < 0.001				
6MWT, m	EG	336.41 (31.14)	392.94 (38.30)	0.95	*F* = 275.987 *p* < 0.001 *ηp* ^2^ = 0.530	*F* = 43.593 *p* < 0.001 *ηp* ^2^ = 0.151	*F* = 33.631 *p* < 0.001 *ηp* ^2^ = 0.121
	CG	332.11 (26.39)	359.39 (21.51)				
	Between group MD (95% CI)	4.30 (–2.94 to 11.53)	33.55 (25.76 to 41.35)				
	*T*	1.170	8.479				
	*p*	0.243	*p* < 0.001				
SF‐36	EG	64.82 (7.48)	77.91 (3.84)	0.97	*F* = 211.151 *p* < 0.001 *ηp* ^2^ = 0.463	*F* = 58.503 *p* < 0.001 *ηp* ^2^ = 0.193	*F* = 34.877 *p* < 0.001 *ηp* ^2^ = 0.125
	CG	62.93 (8.76)	68.45 (9.49)				
	Between group MD (95% CI)	1.89 (–0.15 to 3.93)	9.45 (7.64 to 11.26)				
	*T*	1.823	10.280				
	*p*	0.069	*p* < 0.001				

*Note:* Independent‐*t*‐test; repeated measures ANOVA.

Values are presented as mean (SD); *p < 0.05; **p < 0.01; ***p < 0.001.

Abbreviations: 6MWT, 6‐min walk test; CG, control group; EG, experimental group; MD, mean differences; ROM, range of motion; SF‐36, short form survey‐36.

#### 3.2.1 Primary Outcomes

Pain intensity, NPRS: NPRS scores were comparable between groups at baseline (MD = 0.19; 95% CI: −0.01 to 0.38; *p* = 0.057). Following the intervention, both groups demonstrated reductions in pain levels (EG: 8.05 ± 0.69 to 2.98 ± 1.58; CG: 7.86 ± 0.84 to 4.93 ± 0.95). However, the reduction in pain was significantly greater in the experimental group compared with the control group at post‐intervention (MD = −1.95; 95% CI: −2.28 to −1.62; *p* < 0.001). Consequently, a very large between‐group effect size was observed (Cohen's *d* = 2.79). Repeated‐measures ANOVA revealed highly significant effects for time (*F* = 1851.854, *p* < 0.001, *ηp* ^2 ^= 0.883) and between‐group differences (*F* = 78.571, *p* < 0.001, *ηp* ^2 ^= 0.243). A highly significant group × time interaction was also observed (*F* = 131.968, *p* < 0.001, *ηp* ^2 ^= 0.350), indicating that the magnitude of pain reduction over time differed significantly between the two groups.

Pressure Pain Threshold, PPT: PPT scores were similar between groups at baseline (MD = 0.07; 95% CI: −0.04 to 0.18; *p* = 0.203). After the intervention, both groups showed increases in PPT values (EG: 6.19 ± 0.38 to 7.10 ± 0.22; CG: 6.12 ± 0.48 to 6.64 ± 0.77), indicating reduced pain sensitivity. However, the experimental group demonstrated significantly greater improvement compared with the control group at post‐intervention (MD = 0.46; 95% CI: 0.32–0.60; *p* < 0.001). Consequently, a large between‐group effect size was observed (Cohen's *d* = 0.90). Repeated‐measures ANOVA showed highly significant time effects (*F* = 274.396, *p* < 0.001, *ηp* ^2 ^= 0.529) and between‐group differences (*F* = 32.211, *p* < 0.001, *ηp* ^2^ = 0.117). Additionally, a highly significant group × time interaction was found (*F* = 19.396, *p* < 0.001, *ηp* ^2^ = 0.074), indicating that the increase in PPT over time differed significantly between groups.

Physical function, WOMAC: All WOMAC domains (pain, stiffness, physical function, and total score) were comparable between groups at baseline (WOMAC pain: MD = 0.35, *p* = 0.078; stiffness: MD = 0.18, *p* = 0.186; function: MD = −0.78, *p* = 0.224; total: MD = −0.25, *p* = 0.760). Following the intervention, both groups demonstrated reductions in WOMAC scores, indicating improvements in symptoms and functional status. The experimental group showed greater reductions in WOMAC pain (14.30 ± 1.35 to 6.39 ± 2.02 vs. 13.95 ± 1.71 to 10.17 ± 2.83), stiffness (4.85 ± 1.14 to 1.79 ± 1.22 vs. 4.67 ± 0.99 to 3.44 ± 0.62), physical function (36.30 ± 5.16 to 19.52 ± 2.41 vs. 37.07 ± 4.83 to 26.70 ± 8.41), and total score (55.44 ± 6.23 to 27.69 ± 3.23 vs. 55.69 ± 6.50 to 40.31 ± 9.36) compared with the control group. Between‐group differences after the intervention were statistically significant for all domains (pain MD = −3.78; stiffness MD = −1.65; function MD = −7.18; total MD = −12.62; all *p* < 0.001). Consequently, very large between‐group effect sizes were observed (Cohen's *d* pain = 2.68; stiffness = 1.71; function = 1.28). Repeated‐measures ANOVA demonstrated highly significant time effects for pain (*F* = 1033.448, *ηp* ^2^ = 0.808), stiffness (*F* = 704.309, *ηp* ^2^ = 0.742), function (*F* = 739.445, ηp^2 ^= 0.751), and total (F = 1366.231, *ηp* ^2^ = 0.848); all *p* < 0.001 and also highly significant between‐group effects. Moreover, highly significant group × time interactions were observed for WOMAC pain (*F* = 129.002, *ηp* ^2^ = 0.345), stiffness (*F* = 128.346, *ηp* ^2^ = 0.344), physical function (*F* = 41.176, *ηp* ^2^ = 0.144), and total score (*F* = 112.335, *ηp* ^2^ = 0.314); all *p* < 0.001 indicating that improvements across all WOMAC domains over time were significantly greater in the experimental group compared with the control group.

#### 3.2.2 Secondary Outcomes

Muscle Strength: Knee flexor and extensor muscle strength were similar between groups at baseline (flexor: MD = −2.20; 95% CI: −5.66 to 1.27; *p* = 0.213; extensor: MD = −3.56; 95% CI: −7.65 to 0.54; *p* = 0.089). After the intervention, both groups showed increases in muscle strength; however, the experimental group demonstrated greater improvements (flexor: 120.31 ± 13.73 to 146.85 ± 18.38 vs. 122.51 ± 13.91 to 134.30 ± 12.44; extensor: 139.83 ± 16.38 to 166.43 ± 26.10 vs. 143.39 ± 16.31 to 154.57 ± 16.66). Between‐group comparisons at post‐intervention showed significantly greater strength in the experimental group for both flexor (MD = 12.56; 95% CI: 8.62 to 16.50; *p* < 0.001) and extensor muscles (MD = 11.87; 95% CI: 6.38 to 17.36; *p* < 0.001). Consequently, large between‐group effect sizes were observed (Cohen's *d* flexor = 1.06; extensor = 0.94). Repeated‐measures ANOVA revealed highly significant time effects (flexor *F* = 317.668, *ηp* ^2^ = 0.565; extensor *F* = 143.623, *ηp* ^2^ = 0.370; *p* < 0.001) and group × time interactions (flexor *F* = 47.077, *ηp* ^2^ = 0.161; extensor *F* = 23.933, *ηp* ^2^ = 0.089; *p* < 0.001), indicating that the increase in muscle strength over time differed significantly between groups.

Range of Motion: Knee flexion and extension range of motion (ROM) were comparable between groups at baseline (flexion: MD = 1.33; 95% CI: −1.14 to 3.79; *p* = 0.289; extension: MD = −1.59; 95% CI: −3.34 to 0.16; *p* = 0.074). Following the intervention, improvements in ROM were observed in both groups, with greater changes in the experimental group (flexion: 116.53 ± 10.06 to 134.96 ± 6.91 vs. 115.20 ± 9.59 to 119.23 ± 15.59; extension deficit: 10.77 ± 6.78 to 4.68 ± 5.01 vs. 12.36 ± 7.18 to 8.70 ± 7.10). Between‐group comparisons after the intervention showed significantly greater improvements in the experimental group for both knee flexion (MD = 15.73; 95% CI: 12.71–18.75; *p* < 0.001) and knee extension (MD = −4.02; 95% CI: −5.56 to −2.48; *p* < 0.001). Consequently, very large and small‐to‐moderate between‐group effect sizes were observed (Cohen's d flexion = 1.49; extension = 0.34). Repeated‐measures ANOVA indicated highly significant time effects (flexion *F* = 139.158, *ηp* ^2 ^= 0.362; extension *F* = 225.229, *ηp* ^2^ = 0.479; *p* < 0.001) and group × time interactions (flexion *F* = 57.268, *ηp* ^2^ = 0.189; extension *F* = 14.000, *ηp* ^2^ = 0.054; *p* < 0.001), demonstrating that the magnitude of ROM improvement over time differed between groups.

Waking ability, 6MWT: 6MWT distances were comparable between groups at baseline (MD = 4.30; 95% CI: −2.94 to 11.53; *p* = 0.243). After the intervention, walking distance increased in both groups (EG: 336.41 ± 31.14 to 392.94 ± 38.30; CG: 332.11 ± 26.39 to 359.39 ± 21.51). However, the improvement was significantly greater in the experimental group compared with the control group at post‐intervention (MD = 33.55; 95% CI: 25.76 to 41.35; *p* < 0.001). Consequently, a large between‐group effect size was observed (Cohen's *d* = 0.95). Repeated‐measures ANOVA revealed highly significant effects of time (*F* = 275.987, *p* < 0.001, *ηp* ^2 ^= 0.530) and between‐group differences (*F* = 43.593, *p* < 0.001, *ηp* ^2^ = 0.151). A highly significant group × time interaction was also observed (*F* = 33.631, *p* < 0.001, *ηp* ^2^ = 0.121), indicating that improvements in walking capacity over time differed significantly between groups.

Quality of Life, SF‐36: SF‐36 scores were similar between groups at baseline (MD = 1.89; 95% CI: −0.15 to 3.93; *p* = 0.069). Following the intervention, both groups showed improvements in overall health status (EG: 64.82 ± 7.48 to 77.91 ± 3.84; CG: 62.93 ± 8.76 to 68.45 ± 9.49). However, the improvement was significantly greater in the experimental group compared with the control group (MD = 9.45; 95% CI: 7.64 to 11.26; *p* < 0.001). Consequently, a large between‐group effect size was observed (Cohen's *d* = 0.97). Repeated‐measures ANOVA demonstrated highly significant effects of time (*F* = 211.151, *p* < 0.001, *ηp* ^2^ = 0.463) and between‐group differences (*F* = 58.503, *p* < 0.001, *ηp* ^2^ = 0.193). A highly significant group × time interaction was also identified (*F* = 34.877, *p* < 0.001, *ηp* ^2^ = 0.125), indicating that the improvement in quality of life over time differed significantly between the two groups.

### 3.3 Complications and Adverse Events

Although no serious adverse events were reported. A total of seven participants experienced complications or minor adverse events during the study: four in the experimental group and three in the control group. In the experimental group, two participants reported mild knee soreness and discomfort following exercises, which resolved with rest. Two participants experienced increased knee pain that persisted for a few days but subsided after adjusting the exercise intensity. In the control group, three participants reported mild knee pain, discomfort, and stiffness, which were alleviated with rest and medication adjustments (Table 4). All participants, including those with adverse events, completed the trial without any long‐term consequences.

**Table 4:** Adverse events during the study.

Participant ID	Group	Adverse event	Severity	Action taken	Relationship to intervention
P‐Z12	Experimental	Mild knee soreness	Mild	Short rest; resumed exercise at same intensity	Likely related
P‐Z45	Experimental	Knee discomfort	Mild	Rest; resumed exercise at same intensity	Likely related
P‐Z78	Experimental	Increased knee pain	Moderate	Exercise intensity modified; monitored for 48 h	Possibly related
P‐Z102	Experimental	Temporary knee swelling	Mild	Ice application; exercise resumed at reduced intensity	Possibly related
P‐A07	Control	Mild knee pain/stiffness	Mild	Rest; over‐the‐counter analgesics if needed	Unlikely related
P‐A34	Control	Knee discomfort	Mild	Rest; symptom self‐resolved	Unlikely related
P‐A89	Control	Knee stiffness	Mild	Rest; symptom self‐resolved	Unlikely related

## 4 Discussion

This RCT provides robust evidence that a structured SHEP intervention is an effective and clinically meaningful intervention for individuals with KOA. At post‐intervention, the experimental group demonstrated significantly greater improvements across all outcomes, with 95% confidence intervals that consistently excluded the null value and favored the intervention. Over the 8‐week period, there were significant main effects of time and group, along with a significant group × time interaction, collectively indicating a consistently superior trajectory of improvement in the experimental group across all primary and secondary outcomes. The absence of baseline differences between groups confirms that the observed improvements are attributable to the intervention itself. These findings demonstrate that a well‐designed, strengthening exercise program can produce substantial improvements in pain, physical function, and health‐related quality of life, even when delivered in a self‐managed home‐based context.

Pain intensity, pressure pain threshold (PPT), and physical function were selected as primary outcomes because they collectively represent the central clinical burden of KOA [32]. The experimental group showed a statistically significant and clinically meaningful reduction in NPRS pain scores, surpassing the established MCID for KOA (1.5–2.0 points), indicating a meaningful improvement in pain burden. The effect size was extremely large (Cohen's *d* = 2.79), far exceeding the small‐to‐moderate effects commonly reported in exercise‐based KOA RCTs (approximately Cohen's *d* = 0.40–0.65), suggesting a strong and clinically relevant analgesic response to the intervention [33]. This confirms that the intervention produced perceptible benefits that are relevant to participants' daily experiences. These findings are consistent with previous studies reporting that strengthening exercise programs effectively reduce pain in KOA by improving joint biomechanics and neuromuscular control [10, 34]. The mechanisms underlying pain reduction are multifactorial. Stretching exercises reduce periarticular stiffness and abnormal tensile loading, while strengthening exercises enhance periarticular muscle support and joint stability, thereby decreasing mechanical stress on nociceptive tissues. Improved circulation, synovial fluid distribution, and modulation of inflammatory mediators further contribute to pain relief [33, 34]. These mechanisms are particularly relevant in KOA, where chronic mechanical overload and inflammation interact to sustain pain and disability [35]. Improvements in PPT reflect a meaningful reduction in pain sensitivity and suggest favorable modulation of central sensitization mechanisms, with a large effect size (Cohen's *d* = 0.90) consistent with clinically relevant changes reported in exercise‐based KOA interventions. PPT reflects both peripheral nociceptive sensitivity and central pain processing. Structured strengthening exercise may normalize peripheral nociceptor activity by reducing excessive strain on muscles, tendons, and ligaments, while also inducing central hypoalgesia through enhanced descending inhibitory pathways [33, 34, 35]. Although the change in PPT was highly statistically significant, it did not exceed the previously reported minimal detectable change (MDC = 1.53 kg/cm^2^), suggesting that objective mechanical sensitivity may require a longer or more intensive intervention to achieve clinically detectable improvement. Furthermore, the smaller magnitude of change in PPT compared with subjective pain intensity is consistent with previous evidence indicating that central pain modulation mechanisms may respond more gradually and require greater treatment exposure to produce substantial measurable changes [33, 35, 36]. Physical function assessed by the WOMAC index demonstrated statistically significant and clinically meaningful improvements across pain, stiffness, and function domains in the experimental group, with very large effect sizes (WOMAC pain: Cohen's *d* = 2.68; stiffness: *d* = 1.71; function: *d* = 1.28). These magnitudes substantially exceed the MCID threshold (10–12 points), indicating robust and clinically relevant functional gains. Compared with exercise‐based KOA RCTs, where effect sizes are typically small to very large (approximately Cohen's *d* = 0.25–1.12), the present findings reflect a markedly stronger functional improvement and enhanced patient‐reported outcomes. Strengthening exercises targeting the quadriceps, hamstrings, and hip musculature improve lower‐limb stability and biomechanical efficiency, while additional stretching and ROM exercises reduce periarticular stiffness and facilitate smoother movement. These adaptations restore functional independence and reduce compensatory movement patterns, consistent with prior evidence supporting SHEP intervention as an effective non‐pharmacological intervention for KOA [10, 32, 33, 34, 35].

Secondary outcomes further reinforce the multidimensional benefits of this intervention. The experimental group demonstrated significant improvements in knee flexor and extensor muscle strength, with large effect sizes (Cohen's *d* = 1.06 and 0.94, respectively), exceeding the moderate effects typically reported in exercise‐based KOA RCTs (approximately Cohen's *d* = 0.45–0.75). These gains also exceeded the commonly reported clinical improvement threshold (15%), indicating meaningful functional and physiological adaptations, likely driven by muscle hypertrophy, enhanced motor unit recruitment, and improved neuromuscular coordination [12, 33, 35, 37]. These changes improve joint stability, load distribution, and proprioceptive input, which are critical for both symptom reduction and functional performance [33, 35]. Improvements in knee ROM were clinically meaningful and statistically robust, with a large effect observed for knee flexion (Cohen's *d* = 1.49) and a small‐to‐small/moderate effect for knee extension (Cohen's *d* = 0.34). These gains exceed established MCID thresholds for KOA (flexion: 8°–9°, extension: 3°–4°), indicating functionally relevant improvements in joint mobility that are likely to translate into better gait mechanics and reduced disability. Compared with existing exercise‐based KOA RCTs, where ROM effects are generally small to moderate (approximately Cohen's *d* = 0.20–0.60), the present findings—particularly for flexion—suggest a substantially stronger therapeutic effect, especially for functional flexion capacity. [8, 33, 38]. This structured intervention increases muscle‐tendon compliance, reduces capsular stiffness, and enhances joint compliance, facilitating more efficient movement and safer performance of daily tasks [34, 35, 38]. Walking ability, assessed by the 6MWT, improved significantly with a clinically meaningful gain exceeding the established MCID (26–30 m), indicating enhanced ambulatory endurance and functional mobility. The effect size was large (Cohen's *d* = 0.95), exceeding the small‐to‐moderate effects typically reported in exercise‐based KOA RCTs (approximately Cohen's *d* = 0.40–0.75), suggesting a comparatively strong improvement in walking performance following the intervention. These gains likely result from the combined effects of pain reduction, improved muscle strength, enhanced joint mobility, and better neuromuscular coordination [3, 33, 34]. Improvements in health‐related quality of life across multiple SF‐36 domains were statistically significant and clinically meaningful, exceeding the commonly reported MCID threshold (2–7 points) in populations with KOA, indicating a meaningful improvement in overall health status. The effect size was large (Cohen's *d* = 0.97), which is higher than the small‐to‐moderate effects typically reported in exercise‐based KOA RCTs (approximately Cohen's *d* = 0.21–0.63), suggesting a robust and broad‐based improvement in quality of life outcomes. Reduced pain, improved physical function, and increased mobility directly enhance perceived health status, while the self‐managed nature of the SHEP intervention promotes autonomy, self‐efficacy, and psychological well‐being [12, 33, 34, 35].

The relatively large effect sizes observed across multiple outcomes should be interpreted in light of several study‐specific factors. First, participants demonstrated high baseline symptom severity and functional limitation, providing substantial room for clinical improvement. Second, the intervention combined progressive strengthening, mobility, and flexibility exercises with structured monitoring and behavioral reinforcement, which may have enhanced adherence and therapeutic engagement. Third, the intervention was delivered in a population with limited prior exposure to structured physiotherapy‐based rehabilitation, potentially increasing responsiveness to exercise intervention. Nevertheless, the magnitude of improvement observed in this study was greater than that reported in many previous KOA rehabilitation trials and should therefore be interpreted with caution until replicated in independent populations and in longer‐term studies.

This structured SHEP intervention primarily targeted the neuromuscular and biomechanical deficits associated with KOA, with strengthening exercises as the central component. Exercises focusing on the quadriceps, hamstrings, and hip musculature enhance periarticular muscle strength, which plays a critical role in dynamic joint stabilization, improved load distribution across the knee joint, and reduction of abnormal mechanical stress on degenerated articular cartilage. Improved muscular strength also facilitates more efficient shock absorption during weight‐bearing activities, thereby supporting functional tasks such as walking, stair negotiation, and sit‐to‐stand movements [12, 39]. Complementary joint mobility exercises were incorporated to support the effectiveness of the strengthening component. Light mobility exercises may enhance local circulation, facilitate synovial fluid distribution, and promote joint lubrication while simultaneously supporting neuromuscular activation and proprioceptive awareness, which are essential for maintaining functional stability. Stretching exercises further contribute by improving muscle–tendon flexibility and reducing periarticular stiffness, thereby increasing the knee joint range of motion and allowing smoother, less restricted movement patterns [33, 39]. Collectively, this SHEP intervention, supported by complementary mobility and flexibility exercises, addresses key muscular and joint‐related impairments in KOA. This structured exercise approach improves muscle performance, optimizes joint mechanics, and enhances neuromuscular control, thereby reducing abnormal stress on the joint and lowering pain signals from the affected area. Additionally, it may help regulate pain centrally by activating the body's natural inhibitory pathways, reducing spinal sensitization, and ultimately improving pain, function, and overall joint health in people with KOA [33, 35, 39].

Additionally, despite significant improvements across all measured outcomes, participants did not achieve complete recovery or full symptom resolution on post‐intervention assessments in either the experimental or control group. This incomplete recovery may be attributable to the relatively short 8‐week intervention period, which may be insufficient to produce optimal clinical restoration in individuals with KOA. Additionally, KOA is a multifactorial condition involving pain, muscle weakness, joint stiffness, and functional limitations; therefore, a more comprehensive, well‐structured, multicomponent exercise approach may be required to address these diverse impairments effectively. Consequently, when implementing SHEP intervention for KOA management, consideration should be given to longer intervention durations and the integration of complementary exercise components to optimize therapeutic outcomes.

An important methodological limitation of this trial concerns the unequal intensity of participant contact and engagement across groups. The experimental group received structured exercise instruction, supervised home visits, TheraBand equipment, instructional videos, and ongoing behavioral reinforcement, whereas the control group received pain medication and lifestyle advice. Although both groups were followed up regularly by telephone, the substantially greater level of interaction in the experimental arm may have introduced nonspecific effects, including heightened expectancy, increased motivation, and improved adherence, independent of the specific physiological effects of the exercise intervention. Therefore, the observed between‐group differences favoring the SHEP intervention may, in part, reflect contextual and attention‐related effects rather than the isolated efficacy of the exercise program.

However, these findings should be interpreted with appropriate caution; they may have important contextual implications for Bangladesh and other low‐ and middle‐income countries where access to supervised rehabilitation services is often limited, and healthcare resources remain constrained. The observed effectiveness of a low‐cost, home‐based intervention suggests its potential for broader implementation in community and primary healthcare settings. In such contexts, SHEP intervention may serve as a complementary strategy alongside lifestyle advice, potentially reducing dependence on pharmacological management and facility‐based rehabilitation services. Nonetheless, further implementation‐focused and real‐world effectiveness studies would be needed before drawing firm conclusions regarding large‐scale scalability and health system integration.

### 4.1 Strengths and Limitations

This trial has several notable strengths. The randomized controlled design with assessor blinding enhances internal validity, while the use of validated outcome measures spanning pain, physical function, muscle strength, ROM, walking ability, and quality of life provides a comprehensive evaluation of treatment effects. The inclusion of both physical and psychosocial outcomes allows for a holistic assessment of KOA‐related disability. The application of intention‐to‐treat analysis further strengthens the reliability of the findings. Additionally, the structured, well‐documented SHEP intervention supports reproducibility and facilitates translation into clinical practice. Importantly, this study addresses a critical evidence gap by evaluating a feasible, low‐cost intervention within a real‐world, low‐resource setting. To our knowledge, this is the first large‐scale RCT in South Asia to examine the effects of a structured SHEP intervention in individuals with KOA.

Several limitations should be acknowledged. The study was conducted within a single regional community, which may limit generalizability to other populations or healthcare systems. Adherence monitoring largely relied on participants' log sheets and telephone follow‐ups, and variability in adherence may have influenced treatment effects; unblinded participants may have introduced bias. Although assessor blinding and blinded statistical analysis were implemented, participant and therapist blinding were not feasible due to the nature of the intervention. Furthermore, several major outcomes, including NPRS, WOMAC, and SF‐36, were participant‐reported measures obtained within an unblinded intervention context. Consequently, expectation effects, reporting bias, placebo‐related responses, and differential participant engagement may have influenced outcome reporting, particularly given the greater intensity of therapeutic interaction in the experimental group. The exclusion of individuals with BMI > 30 kg/m^2^, severe deformity, substantial mobility limitations, and limited digital access may also restrict the generalizability of findings to broader KOA populations, particularly those with advanced disease severity, obesity, or limited technological accessibility. The absence of imaging outcomes precludes assessment of structural joint changes. Furthermore, as no adjustment was made for multiple outcome measures, the possibility of an inflated Type I error rate cannot be excluded and should be considered when interpreting the findings. Finally, outcomes were assessed over an 8‐week period, and the long‐term sustainability of benefits remains unknown. Future studies should incorporate extended follow‐up, objective adherence monitoring, telehealth support, and cost‐effectiveness analyses.

## 5 Conclusion

This RCT demonstrated that an 8‐week SHEP intervention was associated with significant improvements in pain, physical function, muscle strength, joint range of motion, walking ability, and health‐related quality of life in individuals with KOA. The observed improvements may be related to enhanced periarticular muscle performance, improved joint stability, improved neuromuscular control, and reduced pain sensitivity, which collectively may facilitate better functional performance and greater participation in daily activities. The intervention was feasible and generally well tolerated by participants throughout the study period, supporting its potential as a practical, accessible rehabilitation approach, particularly in resource‐limited settings where access to supervised physiotherapy services may be limited. These findings highlight the potential role of structured SHEP intervention in extending rehabilitation beyond clinical settings, supporting patient self‐management, and contributing to more accessible and patient‐centered musculoskeletal rehabilitation strategies.

## Author Contributions

**Kazi Md Azman Hossain:** writing – original draft, visualization, methodology, conceptualization, data curation, and project administration. **Jannatul Ferdous Rikti:** writing – review & editing, visualization, data curation, and methodology. **Md. Feroz Kabir:** conceptualization, formal analysis, methodology, and resources. **Sharmila Jahan:** visualization, writing – review and editing, validation, investigation, and resources. **Ehsanur Rahman:** investigation, validation, writing – review and editing, visualization. **K. M. Amran Hossain:** writing – review and editing, validation, methodology, and resources. **Md. Kabir Hossain:** writing – review and editing, data curation, and investigation. **Abid Hasan Khan:** writing – review and editing, funding acquisition, and data curation. **Suraiya Yesmin Sharna:** writing – review and editing, investigation, and data curation. **Md. Zahid Hossain:** writing – review and editing, supervision, conceptualization, project administration, funding acquisition, and validation.

## Ethics Statement

This study was approved by the Institutional Review Board of the Department of Physiotherapy and Rehabilitation, Jashore University of Science and Technology (IRB ID: PTR‐JUST/IRB/2025/02/192404). The study was also prospectively registered in the Clinical Trial Registry of India (ID: CTRI/2025/03/081575). Written informed consent was obtained from all participants prior to enrollment. All procedures and interventions were conducted in accordance with the principles outlined in the Declaration of Helsinki (2020).

## Consent

Consent was obtained from all participants during enrollment.

## Conflicts of Interest

The authors declare no conflicts of interest.

## Transparency Statement

The corresponding author, Md Zahid Hossain, affirms that this manuscript is an honest, accurate, and transparent account of the study reported; that no important aspects of the study have been omitted; and that any discrepancies from the planned study have been explained.

## Supporting information

Supporting File 1

## Data Availability

All data sets generated or analyzed during the current study are available from the corresponding author upon reasonable request.
