# Supplementary material for: Effects of Strengthening‐Based Home Exercise Program on Pain and Function in Knee Osteoarthritis: Phase I Results of a Two‐Phase Randomized Controlled Trial
Source: Health Sci Rep. 2026 Jun 28;9(7):e72705. doi: 10.1002/hsr2.72705 (PMC13311296; doi:10.1002/hsr2.72705)
Supplement: Supplementary file 1 — Supporting File 1 [file HSR2-9-e72705-s001.docx]

**Supplementary File 1. Detailed SHEP Exercise Protocol**

To improve intervention reproducibility and facilitate clinical implementation, a detailed description of the Strengthening-based Home Exercise Program (SHEP) is provided below. The protocol was developed using progressive resistance exercise principles and individualized progression based on participant tolerance and performance capacity. The intervention targeted major lower-limb muscle groups commonly affected in individuals with knee osteoarthritis and was structured to enhance muscular strength, functional performance, and symptom management while maintaining safety and adherence.

# 1. FITT Principle of the SHEP Intervention

| **Component** | **Description** |
| --- | --- |
| Frequency | Three sessions per week for eight-week |
| Intensity | Progressive resistance using color-coded TheraBand resistance levels |
| Time | Approximately 45–55 minutes per session |
| Type | Home-based strengthening exercise program focusing on lower-limb musculature |

# 2. Detailed Exercise Protocol

| **Component** | **Exercise** | **Target Muscle Group** | **Procedure** | **Dosage/**  **Progression** | **Duration/**  **Frequency** |
| --- | --- | --- | --- | --- | --- |
| *Warm-up* | Light walking and marching | General lower-limb activation | Participants performed low-intensity walking and marching at a self-selected pace to increase circulation and prepare the musculoskeletal system for exercise. | Low intensity | 5 minutes/  session |
|  | Active mobility exercises | Hip, knee, and ankle joints | Gentle active range-of-motion exercises were performed for major lower-limb joints. | Controlled movements within a pain-free range | 5 minutes/  session |
| **Strengthening**  **exercise** | Knee flexion-extension with TheraBand | Quadriceps and hamstrings | Exercises were performed in a seated position using elastic resistance bands. | Gradual increase in repetitions and resistance | 10 minutes/  session |
|  | Terminal knee extension | Quadriceps | Participants performed controlled terminal extension movements against elastic resistance. | Gradual increase in repetitions and resistance | 5 minutes/  session |
|  | Leg press | Quadriceps and gluteal muscles | Closed kinetic chain strengthening exercise performed with controlled movement speed. | Gradual increase in repetitions and resistance | 5 minutes/  session |
|  | Calf raise | Gastrocnemius and soleus | Standing heel raise exercise performed with body-weight support as required. | Gradual increase in repetitions and resistance | 5 minutes/  session |
|  | Mini-squat | Quadriceps, gluteals, and core stabilizers | Partial squat movement performed within a pain-free range. | Gradual increase in repetitions and resistance | 5 minutes/  session |
| *Cool-down* | Stretching exercises | Hip flexors, outer hip muscles, quadriceps, and hamstrings | Static stretching was performed following strengthening exercises. | 3 repetitions with 15-second hold | 5 minutes/  session |

# 3. Progressive Resistance Strategy

Exercise intensity was progressively increased across three intervention stages using color-coded TheraBand resistance levels (yellow, red, and green), corresponding to low-, moderate-, and higher-resistance training loads. Progression was implemented to ensure gradual overload adaptation while minimizing symptom exacerbation and maintaining participant safety. Participants advanced through stages based on their exercise tolerance and ability to complete exercises without significant pain exacerbation.

# 4. Safety and Adherence Monitoring

- Participants were instructed to perform all exercises within tolerable pain limits.
- Exercises were discontinued temporarily if severe pain, dizziness, or discomfort occurred.
- Proper posture, breathing technique, and movement quality were reinforced throughout the intervention.
- Participants maintained exercise adherence logs.
- Regular follow-up communication was conducted to monitor compliance and address participant concerns.

# 5. Visual Flowchart of the SHEP Protocol

**Warm-up and Joint Mobility (5 min)
↓
Strengthening Exercises (30 min)
(Knee Flexion–Extension → Terminal Knee Extension → Leg Press → Calf Raise → Mini-Squat)
↓
Progressive Resistance Stages
Stage 1: Yellow Band (Sessions 1–8)
↓
Stage 2: Red Band (Sessions 9–16)
↓
Stage 3: Green Band (Sessions 17–24)
↓
Cool-down Stretching (5 min)**

# 6. Intervention Schedule

The SHEP intervention was administered over an eight-week period with three sessions per week, totaling 24 sessions. Stage 1 was implemented during sessions 1–8, Stage 2 during sessions 9–16, and Stage 3 during sessions 17–24.

*Abbreviation: SHEP = Strengthening-based Home Exercise Program.*
